# Impact of Iron Intake and Reserves on Cognitive Function in Young University Students

**DOI:** 10.3390/nu16162808

**Published:** 2024-08-22

**Authors:** Carmen Dimas-Benedicto, José Luis Albasanz, Laura M. Bermejo, Lucía Castro-Vázquez, Alejandro Sánchez-Melgar, Mairena Martín, Rosa M. Martínez-García

**Affiliations:** 1 NUTRI-SAF Research Group, Departamento de Enfermería, Fisioterapia y Terapia Ocupacional, Facultad de Enfermería, University of Castilla-La Mancha, 16071 Cuenca, Spain; carmen.dimas@alu.uclm.es (C.D.-B.); rosamaria.martinez@uclm.es (R.M.M.-G.); 2 GNCR Research Group, Departamento de Química Inorgánica, Orgánica y Bioquímica, Facultad de Medicina de Ciudad Real, Instituto de Biomedicina de la UCLM, IDISCAM, University of Castilla-La Mancha, 13071 Ciudad Real, Spain; jose.albasanz@uclm.es; 3 VALORNUT Research Group, Departamento de Nutrición y Ciencias de los Alimentos, Facultad de Farmacia, Universidad Complutense de Madrid, 28040 Madrid, Spain; mlbermej@ucm.es; 4 San Carlos Health Research Institute (IdISSC), 28040 Madrid, Spain; 5 NUTRI-SAF Research Group, Departamento de Química Analítica y Tecnología de los Alimentos, Facultad de Farmacia, University of Castilla-La Mancha, 02071 Albacete, Spain; luciaisabel.castro@uclm.es; 6 GNCR Research Group, Departamento de Química Inorgánica, Orgánica y Bioquímica, Facultad de Enfermeria de Ciudad Real, Instituto de Biomedicina de la UCLM, IDISCAM, University of Castilla-La Mancha, 13071 Ciudad Real, Spain; alejandro.sanchez@uclm.es

**Keywords:** iron deficiency anemia, ferritin, dietary iron intake, cognitive scales, female, male, university students

## Abstract

Iron is a key nutrient for cognitive function. During periods of high academic demand, brain and cognitive activity increase, potentially affecting iron intake and reserves. The present study aimed to investigate the impact of iron levels on cognitive function in a university sample, considering the influence of gender. A cross-sectional study was conducted with 132 university students (18–29 years) from the University of Castilla-La Mancha (Spain). A dietary record was formed through a questionnaire to analyze iron consumption, and blood and anthropometric parameters were measured. The Wechsler Adult Intelligence Scale-IV was used to determine the Intelligence Quotient (IQ), as well as the Verbal Comprehension Index (VCI), Working Memory Index (WMI), Processing Speed Index (PSI), and Perceptual Reasoning Index (PRI), to assess cognitive abilities. Among women, the prevalence of iron deficiency (ID) and iron deficiency anemia (IDA) was 21% and 4.2%, respectively. No ID or IDA was found in men. The impact of iron intake on IQ and cognitive abilities was mainly associated with the female population, where a positive association between iron intake, serum ferritin, and total IQ was revealed. In conclusion, low iron intake is related to poorer intellectual ability, suggesting that an iron-rich diet is necessary to maintain the academic level of university students.

## 1. Introduction

Iron deficiency (ID) is one of the most prevalent single-nutrient deficiencies worldwide, primarily affecting children, pregnant women and women of childbearing age, and older people, and it is at least two times more common than iron deficiency anemia (IDA) [1,2]. ID refers to low iron reserves, and ferritin is the most sensitive and specific biomarker for assessing ID [3]. IDA is classified as one of the ten most serious health issues, with a global prevalence of 48% in children aged 6–59 months, 36% in pregnant women aged 15–49 years, and 30% in non-pregnant women aged 15–49 years [4]. ID and IDA are common in both developed and developing countries, and they are often asymptomatic, leading to underdiagnosis. Therefore, their prevalence is estimated to be even higher [5]. Iron is a trace element necessary for oxygen transport (as a component of hemoglobin and myoglobin) and mitochondrial function, among others [6]. Furthermore, iron is a key nutrient for the development and normal functioning of the central nervous system, and it is essential in the processes of myelination, synthesis of neurotransmitters, and the mitochondrial respiratory chain, generating sufficient energy to meet the high neuronal metabolic demand [7]. In line with this, iron and iron-related proteins have been associated with the risk of Alzheimer’s disease (AD) [8], and changes in the iron storage function of ferritin may be related to the pathology of AD and other neurodegenerative diseases [9]. Thus, a link between iron deficiency/anemia and AD has been proposed [10].

IDA arises from a prolonged negative iron balance resulting from reduced intake (sometimes associated with iron-poor vegan or vegetarian diets), blood loss (in women with heavy menstrual bleeding or patients with gastrointestinal disorders), increased iron requirements during periods of rapid growth, or inadequate absorption (calcium, phytates, and tannins decrease absorption, similar to proton pump inhibitors, which increase stomach pH and reduce conversion to ferrous iron [11,12].

Chronic inflammatory disorders such as obesity also influence iron metabolism. Inflammatory cytokines stimulate hepcidin production, leading to decreased intestinal absorption and sequestration of iron in macrophage reserves, reducing transferrin saturation and, consequently, iron availability in tissues [13,14]. Weakness, fatigue, difficulty concentrating, and low productivity are nonspecific symptoms associated with inadequate oxygen supply to body tissues and reduced activity of iron-containing enzymes, although the extent to which these effects manifest before the onset of anemia remains unclear [5].

ID can cause changes in neurotransmitter homeostasis, alter synaptogenesis, decrease myelin synthesis, and affect the function of the basal ganglia (striatum, amygdala, etc.), hippocampus, and prefrontal cortex [15,16]. Therefore, ID can negatively affect cognitive function and ability [17]. IDA has been associated with decreased attention, concentration, reasoning, calculation, speed of response, and memory [18,19], while improvements in cognitive abilities and intelligence quotients have been observed with iron supplementation [15,19,20].

Frequently, iron intake and reserves can be affected during the university period, especially in women of childbearing age [21]. Cognitive demands increase in the university population, and cognitive function may be conditioned by an inadequate supply of nutrients, specifically insufficient iron intake. Considering that ID is the most prevalent deficiency in women of reproductive age, the aim of the study was to assess the influence of iron intake on intellectual and cognitive capacity in female and male university students.

## 2. Materials and Methods

### 2.1. Design and Subjects

In the present study, a cross-sectional design was implemented to recruit university students from the Schools of Nursing, Education, Social Work, Business Administration, Fine Arts, and the Polytechnic School (bachelor’s degree in Telecommunication Technologies Engineering) at the Cuenca campus of the University of Castilla-La Mancha (UCLM), Spain, between June 2018 and December 2019. Sample size calculation was made according to iron deficiency prevalence estimation in the Spanish population [22]. Thus, accepting an alpha risk of 0.95 for a precision of +/−0.08 units in a two-sided test for an estimated prevalence of 2% (in men aged 18–65) and 14% (in women aged 18–52), a minimum of 12 male and 73 female subjects randomly selected from the whole Spanish population was required. To be eligible for participation, students had to meet the following inclusion criteria: (1) be a young university student aged between 18 and 29 years, (2) have no pre-existing pathology mainly related to the nervous, respiratory, digestive, and genitourinary systems, and (3) not be taking medications or toxic substances that could compromise cognitive function. Conversely, students over 29 years old, those with chronic pathologies, and those using medications for depression, anxiety, or substances affecting mood, reaction time, or cognitive ability, including alcohol consumption ≥50 g per week and/or recreational drug use, were excluded. Volunteers who had undergone iron deficiency treatment were also excluded. BMI value was not considered among inclusion or exclusion criteria. The intake of dietary supplements was not considered as an exclusion criterion, since only one woman and two men manifested such intake, and it is unlikely that this low proportion affects the results on cognitive ability.

Informational posters were placed at various faculties and schools to invite participation. The study was conducted after the study protocol was approved by the Ethics Committee of the Virgen de la Luz Hospital in Cuenca (REG: 2017/PI1417; date of approval: 28 May 2018). The study adhered to the ethical standards outlined in the 1964 Declaration of Helsinki and its subsequent amendments. The participants were informed of the study’s rationale and procedures, and they provided informed consent before inclusion. All personal data were confidential and accessible only to the assigned researchers, complying with the European General Data Protection Regulation (2016/679) and Spanish Organic Law of Personal Data Protection (Ley Organica 3/2018).

### 2.2. Study Variables

The selected students, after the submission of signed informed consent, attended two sessions with the researchers at the Nursing Faculty on the Cuenca campus of UCLM. During the first session, conducted within the first week after the delivery of the informed consent, the following variables were collected: personal, socio-demographic, and physical activity data, anthropometric data, and cognitive function data. To collect nutritional data, participants were given a questionnaire to self-record their food consumption over a 4-day period, which they submitted during the subsequent visit. The second session, always within a period not exceeding 20 days from the first visit, involved fasting blood sample extraction for hematological and biochemical analysis and the collection of the previously submitted food consumption questionnaire. The participants had the right to withdraw without explanation at any point during the study.

The methodology employed for collecting these variables is detailed below.

#### 2.2.1. Personal and Socio-Demographic, Lifestyle Data

Personal data were obtained through a questionnaire that recorded name, age, gender, current studies, and place of residence (living with parents, university residences, or student apartments). Regarding health-related data, information on blood pressure, oxygen saturation, resting heart rate, and alcohol and tobacco consumption was collected.

A sphygmomanometer (Boso Manuell model, 0–300 mmHg, Boso, Germany) was used to measure blood pressure, with systolic blood pressure (SBP) defined as the onset of the first arterial sound (start of Korotkoff phase I) and diastolic blood pressure (DBP) as the last audible arterial sound (Korotkoff phase V) [23]. The final data correspond to the average of at least three measurements, with a 5-minute interval between each. The obtained results were compared with the values recommended by the European Society of Hypertension and the European Society of Cardiology [24].

Oxygen saturation [25] and heart rate [26] were measured with a pulse oximeter (Tuffsat Datex Ohmeda model, GE Healthcare, Madrid, Spain). Initially, the fingertip of the subject was massaged before placing the clip with the sensor, avoiding the presence of colorants and pigments in the reading area and ensuring that the fingers were not cold during the procedure.

#### 2.2.2. Physical Activity and Energy Expenditure Data

Physical activity was assessed using data from a validated questionnaire on 24-h activity [27], and the Institute of Medicine (IOM) equations were employed to calculate energy expenditure [28]. The activity questionnaire recorded the time (in minutes) dedicated to each daily life activity with the aim of calculating the activity coefficient for each participant. The time spent on each activity was multiplied by a different factor, according to the WHO guidelines [29]. The result was the individualized activity coefficient, which was substituted with its equivalent using IOM coefficients for the total energy expenditure calculation.

#### 2.2.3. Anthropometric Data

Anthropometric measurements were performed in triplicate in the early morning with the participant barefoot and in lightweight clothing, and the mean value was recorded as the final measurement. Weight was determined using an electronic digital scale (SECA ALPHA model, range: 0.1–150 kg, precision: 100 g, SECA, Hamburg, Germany), and height was measured using a digital stadiometer (model Harpenden, range: 70–205 cm, precision: 1 mm, Holtain Ltd., Crymych, UK) with the participant in an upright position. From these data, body mass index (BMI) (kg/m^2^) was calculated [30], and individuals were classified according to weight status as underweight, normal weight, overweight, or obese [31].

The skinfold thicknesses (bicipital, tricipital, and subscapular) were measured on the non-dominant side of the body using a Holtain Ltd skinfold caliper with a constant pressure of 10 g/mm^2^ of contact surface (range 0–40 mm, precision 0.1 mm). Waist and hip circumferences were measured using an inextensible steel measuring tape (Holtain model) with a range of 0–150 cm, a precision of 1 mm, and expressed in centimeters. Bioimpedance analysis was performed using a single-frequency body composition analyzer (OMRON BF306 Body Fat Monitor, OMRON, Madrid, Spain). The waist-to-hip ratio, waist-to-height ratio, percentage of body fat, and fat-free mass were calculated [32].

#### 2.2.4. Dietary Data

To collect nutritional data, we used a four-day (including Sunday) food and beverage consumption questionnaire previously designed by Ortega et al. [33]. This questionnaire indicates to write down all the food and drinks that are consumed during a certain period of time (3–7 days including a Sunday or holiday). In our case, we wanted to have a greater detail of the dietary pattern (beyond the standard 3-day record); therefore, the participants were requested to fulfill a 4-day record (including 3 weekdays and 1 weekend day). Participants, following instructions from the researchers, recorded all consumed foods both inside and outside the home using weights or household measures. Total food consumption, number of servings, energy and iron intake, and adherence to recommended intake were calculated using Recommended Intake Tables for energy and nutrients for the Spanish population of this age [34]. The dietary analysis employed diet and food data assessment software [35] utilizing Food Composition Tables from the Department of Nutrition and Food Science at the Complutense University of Madrid [36].

To validate the results of the dietary study, the energy intake of each student was compared with the estimated energy expenditure (EE) for each student based on their age, gender, and physical activity. Both values should align when young adults are neither gaining nor losing weight, indicating that they maintain a stable body composition. Otherwise, their intake may have been overestimated or underestimated. The percentage agreement between energy intake and EE was determined using the following equation:

$$Percentage\;Concordance=\left(\frac{Energy\;Expenditure-Energy\;Intake}{Energy\;Expenditure}\right)\times 100$$

A positive result indicates a possible undervaluation of the diet, where the declared energy intake is less than the total estimated energy expenditure. Conversely, a negative value suggests that the declared energy intake exceeds the total energy expenditure, indicating a risk of overestimation [37].

#### 2.2.5. Hematological and Biochemical Data

Blood samples were obtained early in the morning after a 10–12 h overnight fast by puncturing the cubital vein using tubes containing ethylenediaminetetraacetic acid (EDTA) as an anticoagulant for hematological tests and mineral-free tubes for iron and ferritin determination. Red blood cell count, hematocrit index, hemoglobin, red cell distribution width (RDW), and corpuscular values (MCV, MCH, and MCHC) were quantified using a CELL-DYN 29 Plus Calibrator analyzer (Abbott, Madrid, Spain). Serum concentrations of iron and ferritin were measured using a colorimetric method and chemiluminescent microparticle immunoassay (CMIA) method, respectively.

#### 2.2.6. Cognitive Function Study

All cognitive evaluations were conducted post-breakfast and before 2:00 PM. The participants were instructed to have regular breakfast on the cognition test day to ensure comfort and avoid hunger during the session. Cognitive function was assessed using the Wechsler Adult Intelligence Scale-IV [38], which consists of 15 tests grouped into four indices: Verbal Comprehension Index (VCI), Perceptual Reasoning Index (PRI), Working Memory Index (WMI), and Processing Speed Index (PSI). VCI comprises three mandatory tests (similarities, vocabulary, and information) and one optional test (comprehension). The score obtained on this index (70–130) assesses knowledge acquired through education, verbal reasoning, and concept formation and measures aptitudes involving reasoning, comprehension, and conceptualization. The PRI consists of three mandatory tests (blocks, matrices, and visual puzzles) and two optional tests (scales and incomplete figures). The score on this index (70–130) measures visuospatial processing and analysis and visuomotor skills and assesses non-verbal reasoning and perceptual organization. The WMI is comprised of two mandatory tests (digits and arithmetic) and one optional test (letters and numbers). The score on this index (70–130) evaluates the ability to retain and manipulate information in memory while working with it. This index measures simultaneous and sequential processing, attention, and concentration. The PSI comprises two mandatory tests (symbol search and number coding) and one optional test (cancelation). This index assesses the ability to explore, order, and discriminate simple visual information rapidly and efficiently. Mental processing speed and graphomotor skills were measured using this index.

Raw scores from each test were transformed into scaled scores (means and standard deviations) according to the evaluated person’s age group. The scaled scores for Verbal Comprehension, Perceptual Reasoning, Working Memory, Processing Speed, and Total Intelligence Quotient (TIQ) were transformed into composite scores (consulting tables containing percentiles and corresponding confidence intervals for composite score values). These scores represent the values of the respective indices (VCI, PRI, WMI, and PSI), each with a mean of 100 and a standard deviation of 15. This transformation ensures that the data from the four indices and TIQ are comparable.

The TIQ is considered the benchmark index for assessing overall cognitive aptitude [39], as it encompasses various domains of cognitive ability, including verbal comprehension, perceptual reasoning, working memory, and processing speed. Furthermore, it is a global score that condenses an individual’s performance across multiple cognitive aptitudes into a single numerical value (70–130). Testing time ranged from 60 to 90 min, depending on cognitive aptitude, the time needed to maintain a good evaluation environment, and to minimization of subject fatigue.

Regarding cognitive function, and considering the classification of the VCI, PRI, PSI, WMI and TIQ into levels of medium–average (90–109), high-medium (100–119), upper-medium (120–129) and low-medium (80–89), and based on the TIQ score obtained, students were classified into those with medium-high intelligence (TIQ ≥ 90) and low-medium intelligence (TIQ < 90). The sample was also stratified based on the scores of cognitive ability indices (VCI, PRI, PSI, and WMI) as medium-high (scores ≥ 90) or low-medium (scores < 90).

### 2.3. Statistical Analysis

Dietary data were tabulated using DIAL software (for Windows, version 3.0.0.5) [35]. All other sociodemographic, anthropometric, hematological, biochemical, and cognitive capacity-related data were entered into the same database. Data analysis began with descriptive analysis (absolute and relative frequencies, means, and standard deviations). A “z-score” to indicate how many standard deviations a specific value is above or below the mean was calculated with the formula z = σ(X − μ), where X is the individual data point, μ is the mean of the dataset, and σ is the standard deviation of the dataset. A positive z-score indicates that the value is above the mean, whereas a negative z-score indicates that it is below the mean. Statistical analyses included the Student’s *t*-test and the chi-square test. Differences between mean values were considered statistically significant at *p* < 0.05. The D’Agostino and Pearson test was used to check whether values followed a normal distribution. Spearman’s correlation analysis was used to assess the correlation between data variables from different experimental groups. Multiple Linear Regression was conducted to investigate the relationship between dietary iron intake and other covariates (serum hemoglobin, iron, and ferritin) with total IQ. Statistical analysis was performed using the GraphPad Prism 8.0 software (GraphPad Software, San Diego, CA, USA).

## 3. Results

A total of 143 university students (79 women and 64 men) were recruited as the initial sample. Subsequently, 11 participants were excluded either for meeting some exclusion criteria or for not completing the study comprehensively. Finally, 132 young university students, 71 women (53.7%) and 61 men (46.2%), were included in the data processing. Table 1 summarizes the personal, socio-health, and anthropometric data of the young university students, providing an understanding of the sample characteristics.

In men, significantly higher values were observed for systolic blood pressure (SBP) and diastolic blood pressure (DBP), as well as a lower heart rate, compared to women (Table 1); these values are considered optimal according to the European Society of Hypertension and the European Society of Cardiology [24].

Regarding the collected anthropometric variables, most showed significant differences between females and males, with higher weight, height, waist circumference, hip circumference, and fat-free mass in men. 45.9% of male students had excess weight (34.4% overweight and 11.5% obese), and 4% of the female group had low weight (Table 1).

Caloric intake was also significantly higher in men than in women, indicating undervaluation of intake. Although dietary iron intake assessed as a function of caloric intake (mg/Kcal/day) was similar in men and women (Table 2), approximately half of the study population (48.5%) had dietary iron intake below 100% of the recommended intake (RI), with 80.3% of women having iron intake below 100% RI and 39.4% with iron intake < 67% of the RI (Table 2). Lower iron intake in female students was associated with lower consumption of meat, fish, and eggs (2.6 ± 1.1 servings/day) and legumes and cereals (4.0 ± 1.3 servings/day), which are often fortified with iron, compared to males (3.5 ± 1.4 and 5.6 ± 2.2 servings/day, respectively, *p* < 0.001) (Table 3).

When analyzing blood parameters and following the most recent guidelines from the World Health Organization (WHO) published in April 2020, which set a ferritin limit of <15 μg/L for diagnosing iron deficiency (ID) in adults [46], ID was observed in 21% of the female population in the present study, with no ID detected in males. In current clinical practice, ferritin levels < 30 μg/L are identified as mild ID [3], and our study observed that 13.1% of the male population and approximately half (47.9%) of the female population showed mild ID (Table 2).

Regarding iron deficiency anemia (IDA), adopting the limit recommended by the WHO for anemia (Hb < 13 g/dL in men and <12 g/dL in women), a low percentage of female students (4.2%) were found to have levels below the threshold, with no cases of IDA detected among young males (Table 2).

When analyzing serum concentrations, iron and ferritin levels in female students (100.9 ± 42.1 µg/dL and 38.1 ± 26.0 ng/mL) were significantly (*p* < 0.001) lower than in male (114.3 ± 41.3 µg/dL and 109.2 ± 68.5 ng/mL), respectively. ID was observed in 6.6% of the male population and 4.2% of the female population (Table 2).

The average intellectual capacity of the studied population fell within the normal range (90.5 ± 10.8). No significant differences were detected between males with medium-high intelligence (TIQ ≥ 90) and low-medium intelligence (TIQ < 90) when comparing energy and iron intake, hematological and biochemical parameters related to iron status. Similarly, the female group revealed no differences between the TIQ < 90 and TIQ ≥ 90 subgroups (Table 4).

Females with low-medium scores in cognitive abilities measured by VCI, WMI, PRI, and PSI indexes had similar levels of iron intake, coverage of recommended intake, hemoglobin, iron, and ferritin than females with medium-high scores (Table 5). Interestingly, a significant (*p* < 0.01) lower level of serum iron (122.5 ± 40.1 vs. 86.7 ± 33.4 μg/dL) was observed in males with PSI ≥ 90 as compared with PSI < 90, associated with a lower but not significantly lower iron intake (Table 5).

After grouping female and male students according to the recommended intake of iron (%RI), the corresponding hematological and biochemical parameters related to iron status were not statistically different between %RI < 100 and %RI ≥ 100 (Table 6).

**Figure 1:**
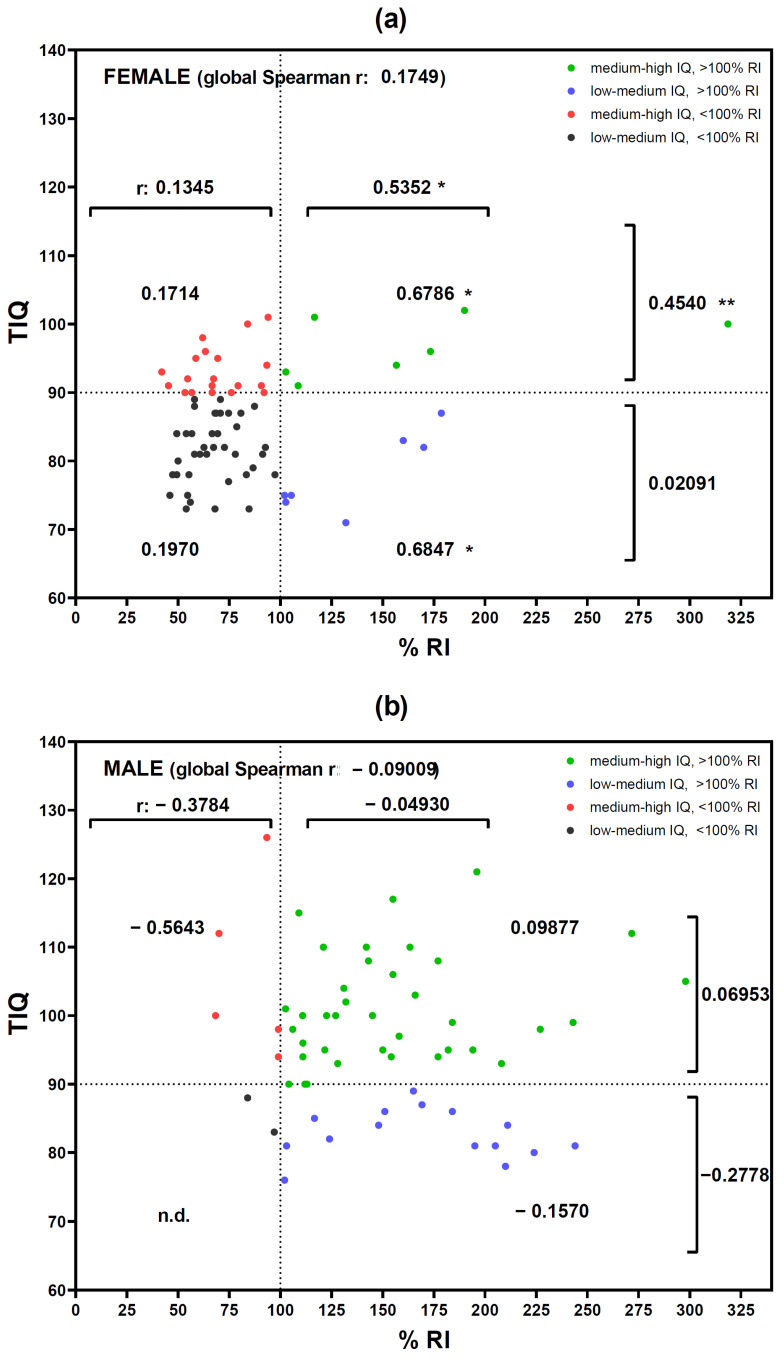
Spearman correlation analysis between TIQ and %RI. Female (**a**) and male (**b**) participants were classified into four groups (low-medium IQ and <100%RI; low-medium IQ and ≥100%RI; medium-high IQ and <100%RI; medium-high IQ and ≥100%RI), and the corresponding r coefficient was calculated. Points represent individual participant’s value. * *p* < 0.05 and ** *p* < 0.01 significant correlation. IQ, intelligence quotient. RI, recommended intake of iron. n.d. not determined.

Students were classified into four subgroups according to their IQ value range (low-medium or medium-high) and their iron intake (<100% RI or ≥100% RI). Blood parameters related to the recommended intake of iron were similar in the four subgroups, except for serum ferritin level in females, which was significantly (*p* < 0.05) higher in young women with medium-high IQ and who had adequate iron intakes vs. young women with low-medium IQ (Table 7). A positive correlation (r = 0.532, *p* < 0.05) was also found between %RI and TIQ in females with %RI higher than 100%, which was even higher when separating this group into low-medium IQ (r = 0.6847, *p* < 0.05) and medium-high IQ (r = 0.6786, *p* < 0.05). Moreover, this positive correlation was also observed (r = 0.4540, *p* < 0.01) in females with medium-high IQ (Figure 1a). A similar analysis in the male group did not reveal this positive correlation (Figure 1b).

**Figure 2:**
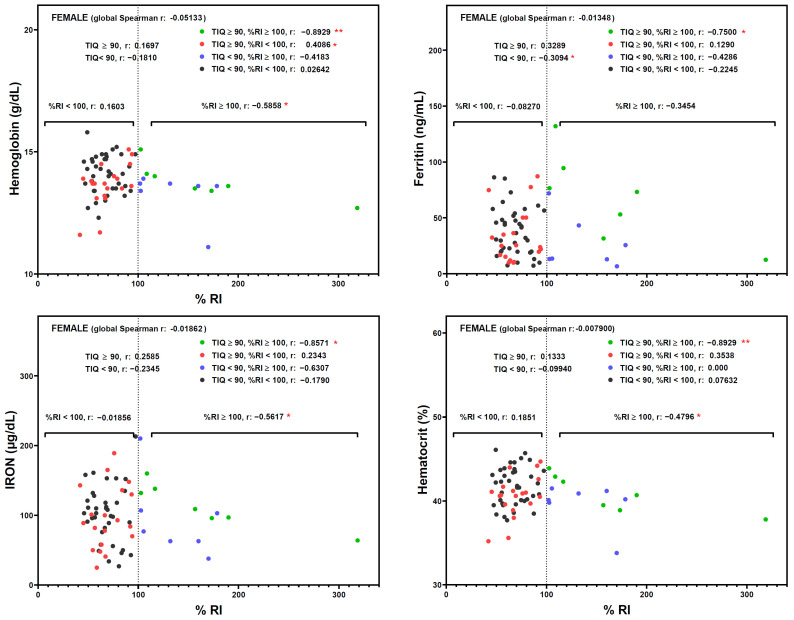
Spearman correlation analysis between TIQ and %RI. Female participants were classified into four groups (low-medium IQ and <100%RI; low-medium IQ and ≥100%RI; medium-high IQ and <100%RI; medium-high IQ and ≥100%RI), and the corresponding r coefficient was calculated. Points represent individual participant’s value. * *p* < 0.05 and ** *p* < 0.01 significant correlation. IQ, intelligence quotient. RI, recommended intake of iron.

**Figure 3:**
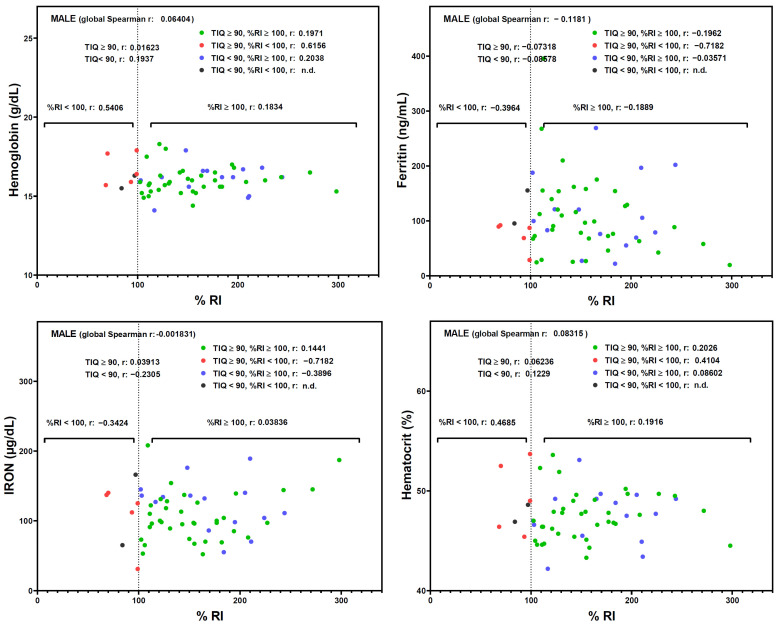
Spearman correlation analysis between TIQ and %RI. Male participants were classified into four groups (low-medium IQ and <100%RI; low-medium IQ and ≥100%RI; medium-high IQ and <100%RI; medium-high IQ and ≥100%RI), and the corresponding r coefficient was calculated. Points represent individual participant’s value. IQ, intelligence quotient. RI, recommended intake of iron. n.d. not determined.

The %RI also influenced several biochemical markers of iron status in females (Figure 2). Of note, a negative correlation between %RI and hemoglobin (r = −0.8929), ferritin (r = −0.7500), iron (r = −0.8571), and hematocrit (r = −0.8929) was detected in the subgroup of female students with TIQ ≥ 90 and %RI ≥ 100, suggesting a detrimental effect when excessive amounts of iron are ingested. In male subgroups, no significant correlations were detected (Figure 3).

**Figure 4:**
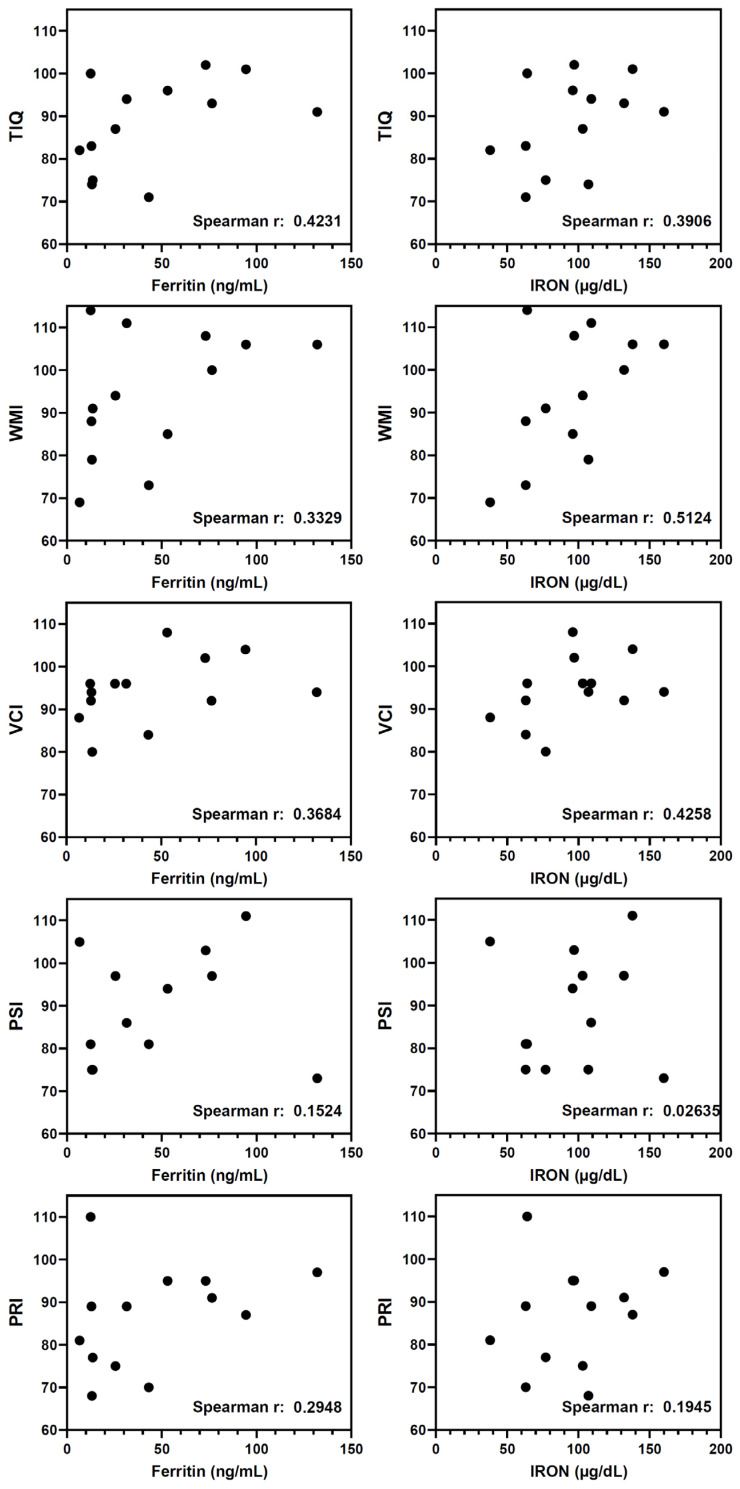
Spearman correlation analysis between serum markers (ferritin and iron) and cognitive indices (TIQ, WMI, VCI, PSI, PRI). The r coefficient is shown within de corresponding graph. Points represent individual participants’ values of the female group with iron intake higher than 100% of the recommended intake.

The possible correlation between biochemical markers of iron status and cognitive indices was also analyzed in the different subgroups. In females with a %RI higher than 100%, a positive correlation was detected between ferritin or iron levels and these cognitive indices, mainly TIQ, WMI, and VCI (Figure 4). Again, no correlations were detected in the male group.

Finally, a multiple linear regression (MLR) analysis of the relationship between dietary iron intake (independent variable) and other covariates (serum hemoglobin, iron, and ferritin) with total IQ (dependent variable) revealed a positive association of total IQ with iron intake (%RI) in female group (Table 8), while no significant associations were observed in male group (Table 9). Since the regression coefficients (ß_1_, …, ß_k_) may be in different measurement units, a direct comparison is difficult. Therefore, the standardized regression coefficients were calculated to eliminate this problem by expressing the coefficients in terms of a single, common set of statistically reasonable units so that comparison may at least be attempted. Thus, a comparison of the absolute values of the standardized regression coefficients suggests the relative importance of the variables, dietary iron intake (%RI), the most important variable in the overall female group (ß_1_ = 1.926, *p* = 0.04) and the female with TIQ ≥ 90 group (ß_1_ = 1.428, *p* = 0.01).

## 4. Discussion

Results presented herein are suggestive of a relationship between iron status and intellectual capacity in young female students. Our study reveals that the iron nutritional status of the university population is suboptimal, with 48.5% of the studied population having diet iron intake below the recommended intake (RI). When assessing the impact of iron on IQ and cognitive abilities, it was observed that the effect of iron intake on IQ was mainly associated with the female population.

Health and anthropometric data revealed that the study group consisted of healthy university students, with a high percentage (45.9%) of males exhibiting excess weight similar to the 50% reported by Iglesias et al. in young university students from Madrid (Spain) [47], but higher than the 20% reported by Gallo et al. in a group of Australian biomedicine male students [48] or the 28.5% and 22.5% reported by Martinez Roldan et al. [49] and García-Meseguer et al. [50] respectively, in Spanish university students.

In general, the diet of female university students is slightly hypocaloric, with an energy intake similar to that observed in other studies [48,49,50,51]. Although their caloric consumption was significantly lower than that of males, females, and males present a similar contribution to recommended energy intake. This may be because, although males consume more energy than females, their energy expenditure is greater than that of female students.

More than half of the studied group (62.1%) had energy intakes lower than the theoretical caloric expenditure. This may indicate some degree of undervaluation of intake in some students, mainly in the male group (3%), a trend observed in other groups of young students [37,52]. It is also possible that many university students restrict their energy intake because of fear of gaining excess weight, a situation observed by other authors [37,53,54].

Dietary iron intake in young female students (12.7 ± 6.5 mg/day) was lower than that observed by Gallardo-Escudero et al. (19.1 ± 1.0 mg/day) in a cohort of young female university students in Granada (Spain) [55], similar to that reported (12.1 ± 4 mg/day) by Beaudry et al. in young female students at Brock University (Canada) [51], and higher than that reported (8.8 mg/day) by Gallo et al. in Australian university female students [48], as well as being above the median (9.8 mg/day) observed in a group of women from a representative sample of the Spanish population (ANIBES study) [56].

The recorded iron intake corresponds only to the intake of food and beverages. Given that only 1.5% of the study group took nutritional supplements with iron, it is unlikely that supplement use had a significant impact on iron intake. It is important to mention that a high percentage of females (80.3%) did not reach the RI, and almost half (39.4%) showed iron intakes below 67% of the RI. These percentages are higher than those (43.6% and 23.6%, respectively) found by Gallardo-Escudero et al. [55] in young women. The lower iron intake in our studied female population may be attributed to the reduced consumption of meat, fish, and eggs, which is lower than that observed by other authors [57], as well as cereals (enriched with iron) and legumes compared to male students and failing to meet the recommended 6–10 servings/day of cereals and legumes in both sexes [58]. Insufficient iron intake in the female population is a concern, given the importance of this mineral in fertility, physical health, and cognitive function [15,16,18,19,59].

Serum iron values were similar to those obtained in other studies [47,55]. Regarding iron deficiency states based on ferritin levels of less than 15 ng/mL, only young women presented ID (21%), a prevalence lower than that observed by Fayet-Moore et al. (33.9%) in university women [60], similar to the 20% reported by Cook et al. in young women aged 18–35 [61] and the 22.7% observed by Ortega et al. in a group of Spanish female adolescents [62], and higher than the 9.7% reported by Milman et al. in young women of similar ages to the studied population [63]. ID in female university students may be associated with loss due to menstruation and low iron intake [12,13], which may contribute to the significantly lower ferritin level than that observed in our male group. It is worth noting that almost half of the female university students (47.9%) had mild ID, which is a concern given that ferritin levels < 30 µg/L have been associated with infertility [59]. Additionally, it is important to detect ID before the onset of pregnancy to avoid the risks of perinatal ID or IDA, which include neurocognitive effects and low birth weight of the offspring [64]. In addition, 13.1% of male students had mild ID, a situation that may be attributed to lower iron absorption due to their excess body weight [13,14].

Regarding hematological parameters [44,45], the number of red blood cells, as well as the concentrations of hemoglobin and hematocrit, were similar to those found in other groups of university students [47,55]. The prevalence of IDA in the female studied population (4.2%) was a little higher than that found (2.3%) by Ibañez-Alcalde et al. in Spanish female adolescents aged 12–16 [65]. Similar results were observed elsewhere in Europe, such as the 2.2% reported by Milman et al. in young Danish women [63], and in other countries world-wide, as noted by Fayet-Moore et al. [60] and Cook et al. [61] in young Australian university women (3% and 6%, respectively), or the 2.1% reported by Grille et al. [66] in university women in Uruguay and the 8% observed by Saxena et al. [67] in Himalayan university female students. However, the percentage of IDA in our female group was lower than the 25.4% reported by Rani et al. [68] in a cross-sectional study in India and the 14.3% reported by Amoaning et al. [69] in Ghana female university students.

Iron intake could influence blood serum levels of hemoglobin, hematocrit, and ferritin. However, no significant differences were found between individuals of the same gender taking less or more than 100% RI of iron (Table 6). Insufficient iron intake, as well as deficient serum levels of iron, hemoglobin, and ferritin, should be avoided in the general population and especially in young students, given their potential relationship with decreased cognitive capacity and academic performance. Iron is a key nutrient for neural function and is essential in the processes of myelination and synthesis of neurotransmitters, primarily serotonin and catecholamines [15,16,18,19].

Petranovic et al. have suggested that cognitive performance is strongly related to hemoglobin levels in both anemic and non-anemic individuals [70]. It has been reported that a decrease of 1 g/dL in hemoglobin is associated with a decrease of 1.73 points in IQ in children [71]. In our study we did not find a positive correlation between hemoglobin levels and TIQ scores in the female or male group, contrary to reported by Khedr et al. [72].

A study conducted by More et al. observed a relationship between hemoglobin levels and TIQ in adolescent girls in India aged 12–15 since the TIQ of girls with IDA (96.6 ± 7.2) was significantly lower than it was for those without IDA (107.8 ± 4.9) [73]. In our study, when female and male groups were analyzed separately, mean values of iron intake, as well as ferritin and hemoglobin levels, were not different in medium-low (<90) from those with medium-high (≥90) TIQ. However, serum ferritin levels were higher in women with higher intellectual capacity vs. those with lower intellectual capacity, presenting adequate intakes in both groups at the time of the study. In addition, a positive correlation between iron intake (%RI) and TIQ was observed in females when separated into four subgroups attending to their corresponding IQ (above or below 90) and %RI range (above or below 100%). This correlation was not observed in the male population, in which high iron intake values were predominant.

Regarding the cognitive abilities of young students assessed through the VCI, WMI, PRI, and PSI scales and their correlation with biochemical markers of iron status, our study revealed a positive correlation between ferritin or iron levels and these cognitive indices, mainly TIQ, WMI and VCI, in those females with iron intake higher than 100% of the recommended intake. Similar results were observed by Sen and Kanani in a group of young adolescents using the Wechsler Intelligence Scale for Children (WISC) for cognitive assessment, finding significantly lower scores in the visual memory and digits test in anemic girls than in non-anemic [74]. Ortega et al. also demonstrated an association between ferritin levels and attention (r = 0.3383, *p* < 0.05) and hemoglobin levels and calculation ability (r = 0.2905, *p* < 0.05) in a group of adolescents aged 15–18 [62]. Similarly, More et al. [73] observed that scores on tests of attention, concentration, and visual memory decreased in a group of adolescent girls with ID, both anemic and non-anemic, and that iron supplementation improved scores in these cognitive abilities. The effects of iron supplementation on cognitive scores were also evident in a meta-analysis by Falkingham et al. [20], based on 14 randomized controlled trials, mostly involving children or adolescents, where iron supplementation improved attention and concentration regardless of the initial iron level (standardized mean difference 0.59; 95% CI: 0.29 to 0.90). Additionally, in the anemic groups, supplementation improved IQ by 2.5 points (95% CI: 1.24 to 3.76) but did not affect non-anemic participants’ IQ, memory, psychomotor skills, or school performance. In line with this, our results revealed a positive correlation between TIQ and %RI (r = 0.3775, *p* < 0.0001), serum ferritin (r = 0.2753, *p* = 0.0014), and hemoglobin (r = 0.3373, *p* < 0.001), considering the whole population. A meta-analysis by Low et al. [75] in children aged 5 to 12 years old showed that iron supplementation improved attention, concentration, and IQ scores in anemic groups (standardized mean difference 4.55; 95% CI: 0.16 to 8.94, *p* = 0.04). A recent systematic review by Samson et al. [76] based on 50 studies (26 cross-sectional and 24 iron intervention studies) also suggested that iron levels and IDA may be associated with academic performance and that iron supplementation during adolescence can improve school performance, attention, and concentration. However, the authors noted that almost all the supplementation trials had a moderate or high risk of bias. No evidence was found to suggest that iron levels and IDA influence, or are associated with, intelligence or memory in adolescents. Additionally, iron supplementation did not improve memory, recall, or intelligence, so further research is needed to improve understanding of the relationship between anemia and educational performance. However, the main limitation of this review is that the authors were unable to conduct a meta-analysis because of different outcome measures among studies and diversity in the ages of the samples.

The strengths of the present study include the assessment of iron intake obtained through a four-day food consumption record, which included a holiday, and the utilization of the Wechsler Adult Intelligence Scale-IV to study cognitive function. Additionally, to the best of our knowledge, this is the first study conducted among young Spanish university students to analyze iron intake based on dietary records and its impact on both IQ and the cognitive abilities of the participants. This study will serve to establish considerations regarding the iron status and cognitive performance of university students. One of the main limitations of the present study is that it was conducted with a relatively small sample size from a single Spanish university. Therefore, despite the iron nutritional status being like that found in other studies, it probably does not adequately represent the Spanish population, making it challenging to robustly assess the effect of this mineral on cognitive function. Another limitation is the cross-sectional design of the study, which did not allow for the establishment of causal relationships. Follow-up or interventional studies should be conducted to assess whether cognitive improvements occur with an increase in iron nutritional status. Despite these limitations, the findings of this study should serve as a starting point for further exploration of the impact of the nutritional status of this mineral on cognitive performance.

## 5. Conclusions

Our data highlight the close relationship between iron nutritional status, as well as certain parameters related to iron, and the cognitive performance of the university population, mainly the female population. Identifying young individuals with inadequate iron intake, as well as those with deficient serum and hematological levels, and then improving their iron status should be a health and educational priority, contributing to the enhancement of the cognitive abilities and academic performance of the students. Well-designed interventional studies are needed to solidify the scientific evidence regarding the effects of improving the nutritional status of this mineral and, therefore, cognitive performance.

## Figures and Tables

**Table 1:** Personal, health, and anthropometric data. Differences based on gender.

	Female	Male
**Personal and Lifestyle data**		
Age (years)	19.7 ± 1.6	21.2 ± 2.6 ***
Place of cohabitation (%)		
Living with parents	13.4	29.5
Student flat	71.6	52.5
Student residence	14.9	18
Smoking habit (%)		
Non-smoker	67.6	78.7
Ex-smoker	12.5	10.4
Smoker	32.4	21.3
Cigarettes/day	6.0 ± 4.8	6.6 ± 4.9
Alcohol consumption (g/day)	4.1 ± 9.2	5.5 ± 13.1
**Health data**		
Systolic blood pressure (mmHg) (normal values) ref. [24]	96.5 ± 11.3 (120–129)	108.4 ± 10.6 *** (120–129)
Diastolic blood pressure (mmHg) (normal values) ref. [24]	60.7 ± 10.8 (80–84)	68.7 ± 9.7 *** (80–84)
Oxygen saturation (%) (normal values) ref. [25]	97.2 ± 1.5 (>95)	96.4 ± 4.6 (>95)
Heart rate (beats/min) (normal values) ref. [26]	84.8 ± 13.0 (60–90)	80.0 ± 12.6 * (60–90)
**Anthropometric data**		
Weight (kg)	59.2 ± 8.9	76 ± 10.9 ***
Height (cm)	161.7 ± 5.7	174.6 ± 7.0 ***
BMI (kg/m^2^)	22.6 ± 3.0	25.0 ± 3.9 ***
Weight status (%) ref. [31]		
Underweight (BMI < 18.5)	4.2	0.0
Normal weight (BMI: 18.5–24.9)	77.5	54.1
Overweight (BMI: 25.0–29.9)	16.9	34.4
Obese (BMI ≥ 30.0)	1.4	11.5
Waist circumference (cm)	75.9 ± 8.7	88.2 ± 9.4 ***
cardiometabolic risk, ref. [31]		
Very low	(<80)	(<94)
Incremented	(80 to 87)	(94 to 101)
High	(≥88)	(≥102)
Hip circumference (cm)	96 ± 13.87	100 ± 8.15 *
cardiometabolic risk ref [40]		
Very low	(<80)	(<94)
Incremented	(80 to 88)	(94 to 102)
High	(>88)	(>102)
Waist-to-hip ratio	0.89 ± 0.91	0.88 ± 0.05
cardiometabolic risk ref [41]		
Substantially increased	(≥0.85)	(≥0.90)
Waist-to-height ratio	0.47 ± 0.05	0.51 ± 0.06
high risk of lifestyle-related disorders ref [42]	(≥0.5)	(≥0.5)
Bicipital fold (mm)	10.2 ± 4.5	8.5± 4.9 *
Tricipital crease (mm)	16.6 ± 10.0	12.8 ± 6.8 *
Subscapular fold (mm)	17.0 ± 7.3	17.0 ± 6.7
Body fat (%)	32.3 ± 5.40	23.3 ± 7.18
reference values ref [43]		
Limit	(31–33%)	(21–25%)
Obesity	(>33%)	(>25%)
Fat-free mass (%)	68 ± 5.40	77 ± 7.18 ***

Data are means ± standard deviation of female (n = 71) and male (n = 61) participants. * *p* < 0.05 and *** *p* < 0.001 significantly different from the corresponding female value. BMI: body mass index.

**Table 2:** Energy and iron intake, and other blood parameters related to iron status. Differences based on gender.

	Female	Male
Energy (Kcal/day)	1881.8 ± 332.7	2550.1 ± 587.7 ***
RI (Kcal/day)		
14–19 years	2250	2800
20–39 years	2200	2700
RI Contribution (%)	99.6 ± 18.8	97.0 ± 24.9
<100% RI (%)	64.8	59.0
<67% RI (%)	2.8	9.8
Energy Expenditure (Kcal/day)	1906.4 ± 196.6	2654.6 ± 202.1
Underestimation (%)	0.4	3.0
Iron intake (mg/day)	12.7 ± 6.5	17.5 ± 10.9 **
Iron intake (mg/Kcal/day)	6.8 ± 3.6	6.7 ± 2.7
RI (mg/day)		
14–19 years	15	12
20–39 years	15	10
RI Contribution (%)	84.5 ± 43.6	167.6 ± 109.1 ***
<100% RI (%)	80.3	11.5
<67% RI (%)	39.4	0.0
**Hematology**		
Red blood cells (mill/µL) (normal values) ref. [44]	4.7 ± 0.3 (3.5–5.0)	5.4 ± 0.30 *** (4.3–5.9)
Hemoglobin (g/dL) (normal values) ref. [45] IDA (%) (cut-off reference value)	13.9 ± 0.9 (11.7–15.5) 4.2 (<12 g/dL)	16.1 ± 0.9 *** (13.2–17.3) 0.0 (<13 g/dL)
Hematocrit (%) (normal values) ref. [45] Deficiency (%)	41.2 ± 2.4 (33–43) 4.2	47.7 ±2.6 (39–49) 0.0
MCV (µm^3^) (normal values) ref. [44]	88.5 ± 4.4 (86–98)	88.9 ± 3.0 (86–98)
MCH (pg) (normal values) ref. [44]	29.8 ± 1.7 (27–32)	30.0 ± 1.1 (27–32)
MCHC (%) (normal values) ref. [44]	33.7 ± 0.5 (33–37)	33.6 ± 0.7 (33–37)
RDW (%) (normal values) ref. [44]	14.7 ± 1.8 (11–18)	13.2 ± 1.2 *** (11–18)
**Biochemistry**		
Serum ferritin (ng/mL)	38.1 ± 26.0	109.2 ± 68.5 ***
(normal values) ref. [3]	(10–130)	(27–300)
Mild ID, ferritin < 30 ng/mL (%)	47.9	13.1
ID, ferritin < 15 ng/mL (%)	21.1	0.0
Serum iron (µg/dL)	100.9 ± 41.8	114.3 ± 41.3
(normal values) ref. [44]	(60–160)	(80–180)
Deficiency (%)	4.2	6.6
(cut-off reference value)	(<37 µg/dL)	(<59 µg/dL)

Data are means ± standard deviation of female (n = 71) and male (n = 61) participants. ** *p* < 0.01 and *** *p* < 0.001 significantly different from female value. RI, recommended intake; MCV, mean corpuscular volume; MCH, mean corpuscular hemoglobin; MCHC, mean corpuscular hemoglobin concentration; RDW, red cell distribution width; ID, iron deficiency. IDA, iron deficiency anemia.

**Table 3:** Consumption of food (in servings) among young university students. Differences based on gender.

Food Group	Female	Male
Cereals and pulses	4.0 ± 1.3	5.6 ± 2.2 ***
Vegetables	2.1 ± 1.1	3.1 ± 1.5 ***
Fruits and derivatives	1.1 ± 1.2	1.5 ± 1.1
Meat, fish and eggs	2.6 ± 1.1	3.5 ± 1.4 ***
Dairy products	1.5 ± 0.8	2.3 ± 1.7 ***

Data are means ± standard deviation of Female (n = 71) and Male (n = 61) participants. *** *p* < 0.001 significantly different from the corresponding female value.

**Table 4:** Energy and iron intake, hematological and biochemical parameters related to iron status. Differences based on intelligence quotient.

	Female		Male	
	TIQ < 90 (n = 45)	TIQ ≥ 90 (n = 26)	TIQ < 90 (n = 17)	TIQ ≥ 90 (n = 44)
Energy (Kcal/day)	1867.0 ± 314.1	1907.5 ± 367.7	2443.6 ± 582.5	2591.2 ± 591.2
Contribution RI (%)	99.6 ±18.4	99.6 ± 19.7	91.6 ± 24.5	99.0 ± 25.0
Under/Over-estimated	0.4 ± 18.4	0.4 ± 19.7	8.4 ± 24.5	1.0 ± 25.0
Iron (mg/day)	11.7 ± 4.6	14.3 ± 8.8	16.7 ± 5.3	17.9 ± 12.4
Contribution RI (%)	78.1 ± 30.7	81.6 ± 31.7	160.8 ± 50.0	170.2 ± 125.1
**Hematology**				
Red cells (mill/µL)	4.7 ± 0.3	4.6 ± 0.3	5.3 ± 0.3	5.4 ± 0.3
Hemoglobin (g/dL)	14.0 ± 2.2	13.7 ± 0.8	16.0 ± 0.9	16.1 ± 0.9
Hematocrit (%)	41.5 ± 6.6	40.6 ± 2.4	47.6 ± 2.6	47.7 ± 2.6
MCV (µm^3^)	88.9 ± 3.5	87.7 ± 5.6	89.5 ± 2.5	88.6 ± 3.2
MCH (pg)	30.0 ± 1.5	29.5 ± 2.1	30.2 ± 1.1	29.9 ± 1.1
MCHC (%)	33.7 ± 0.5	33.6 ± 0.5	33.6 ± 0.7	33.6 ± 0.7
RDW (%)	14.6 ± 1.1	14.8 ± 2.7	13.5 ± 0.9	13.1 ± 1.2
**Biochemistry**				
Ferritin (ng/mL)	35.5 ± 22.1	42.6 ± 32.2	115.6 ± 66.8	106.8 ± 69.8
Mild ID, ferritin < 30 ng/mL (%)	48.9	46.2	11.8	13.6
ID, ferritin < 15 ng/mL (%)	22.2	19.2	0.0	0.0
Iron (µg/dL)	100.9 ± 44.2	101.0 ± 42.0	121.8 ± 38.4	111.4 ± 42.5
Deficiency (%)	4.4	3.8	5.9	6.8

Parameters from students with low-medium (<90) and medium-high (≥90) total intelligence quotient (TIQ) scores. Data are means ± standard deviation of indicated n participants. RI, recommended intake; MCV, mean corpuscular volume; MCH, mean corpuscular hemoglobin; MCHC, mean corpuscular hemoglobin concentration; RDW, red cell distribution width; ID, iron deficiency. Iron deficiency, iron < 37 µg/dL (female) or 59 µg/dL (male).

**Table 5:** Iron intake and blood parameters related to iron status in relation to indices assessing cognitive abilities. Differences based on cognitive indices.

	Verbal Comprehension Index (VCI)		Working Memory Index (WMI)		Perceptual Reasoning Index (PRI)		Processing Speed Index (PSI)	
**Female**	**<90** **(n = 26)**	**≥90** **(n = 45)**	**<90** **(n = 35)**	**≥90** **(n = 36)**	**<90** **(n = 41)**	**≥90** **(n = 30)**	**<90** **(n = 41)**	**≥90** **(n = 30)**
Iron (mg/day)	11.5 ± 4.1	13.3 ± 7.6	11.9 ± 4.9	13.4 ± 7.8	12.3 ±5.2	13.1 ± 8.1	11.9 ± 7.1	13.7 ± 5.7
% RI	76.8 ± 27.1	88.9± 50.6	79.3 ± 32.9	89.5 ± 52.0	82.2 ± 34.6	87.6 ± 54.2	79.5 ± 47.1	91.4 ± 38.1
Iron (μg/dL)	104.4 ± 42.1	98.9 ± 41.9	99.9 ± 45.1	102.0 ± 38.9	96.7 ± 37.8	106.7 ± 46.7	98.2 ± 42.0	104.6 ± 41.9
Hb (g/dL)	13.9 ± 1.0	13.8 ± 0.8	13.9 ± 0.9	13.8 ± 0.9	13.8 ± 0.8	13.9 ± 0.9	13.9 ± 0.9	13.9 ± 0.9
Ferritin (ng/mL)	37.3 ± 21.1	38.6 ± 28.7	35.4 ± 22.8	40.7 ± 28.9	34.1 ± 23.0	43.6 ± 29.1	39.5 ± 26.1	36.2 ± 26.2
**Male**	**<90** **(n = 1)**	**≥90** **(n = 60)**	**<90** **(n = 9)**	**≥90** **(n = 52)**	**<90** **(n = 26)**	**≥90** **(n = 35)**	**<90** **(n = 47)**	**≥90** **(n = 14)**
Iron (mg/day)	21.1 ± n.d	17.5 ± 11.0 *	14.0 ± 4.7	18.1 ± 11.6 *	16.9 ± 7.8 **	18.0 ± 12.8	18.7 ± 12.0 **	13.5 ± 4.0
% RI	211.0 ± n.d.	166.8 ± 109.8 ***	134.5 ± 48.5 ***	173.3 ± 115.8 ***	162.3 ± 77.6 ***	171.5 ± 128.5 **	180.3 ± 120.1 ***	124.8 ± 36.1 **
Iron (μg/dL)	70.0 ± n.d.	115.0 ± 41.3	105.9 ± 34.6	115.7 ± 42.5	115.3 ± 34.8 *	113.5 ± 46.1	122.5 ± 40.1 **	86.7 ± 33.4 ††
Hb (g/dL)	15.0 ± n.d.	16.1 ± 0.9 ***	16.0 ± 0.5 ***	16.1 ± 0.9 ***	16.3 ± 0.9 ***	16.0 ± 0.9	16.1 ± 0.9 ***	16.0 ± 0.9 ***
Ferritin (ng/mL)	105.6 ± n.d.	109.3 ± 69.1 ***	96.4 ± 58.3 ***	111.5 ± 70.4 ***	115.1 ± 69.2	104.9 ± 68.7 ***	110.5 ± 75.1 ***	105.0 ± 40.9 ***

Parameters from students with low-medium (<90) and medium-high (≥90) scores on indicated cognitive indices (VCI, WMI, PRI, and PSI). Data are means ± standard deviation of indicated n participants. * *p* < 0.05, ** *p* < 0.01 and *** *p* < 0.001 significantly different from female value. †† *p* < 0.01 significantly different from the corresponding “<90” group. RI, recommended intake; Hb, hemoglobin. n.d., not determined.

**Table 6:** Energy and iron intake, hematological and biochemical parameters related to iron status. Differences based on recommended intake of iron.

	Female		Male	
	%RI < 100 (n = 57)	%RI ≥ 100 (n = 14)	%RI < 100 (n = 7)	%RI ≥ 100 (n = 54)
Energy (Kcal/day)	1829.1 ± 361.1	2096.7 ± 464.4 *	1969.3 ± 550.4	2625.4 ± 553.6 **
Contribution RI (%)	96.3 ±19.1	113.2 ± 27.3 **	75.1 ± 23.9	99.8 ± 23.8 *
Under/Over-estimated	3,7 ± 14,3	−13.2 ± 27.3 **	24.9 ± 23.9	0.2 ± 23.8 *
Iron (mg/day)	10.2 ± 2.5	22.7 ± 8.7 ***	9.4 ± 1.1	18.6 ± 11.2 *
Contribution RI (%)	68.1 ± 16.9	151.2 ± 58.1 ***	87.2 ± 13.4	178.0 ± 111.7 *
**Hematology**				
Red cells (mill/ µL)	4.7 ± 0.7	4.6 ± 0.2	5.5 ± 0.3	5.4 ± 0.3
Hemoglobin (g/dL)	13.9 ± 2.0	13.5 ± 0.9	16.5 ± 1.0	16.0 ± 0.9
Hematocrit (%)	41.4 ± 5.9	40.3 ± 2.4	48.9 ± 3.1	47.5 ± 2.5
MCV (µm^3^)	88.5 ± 12.5	88.3 ± 3.2	89.3 ± 3.2	88.8 ± 3.0
MCH (pg)	29.8 ± 4.3	29.7 ± 1.3	30.1 ± 1.1	20.0 ± 1.1
MCHC (%)	33.7 ± 4.5	33.6 ± 0.5	33.7 ± 0.6	33.6 ± 0.7
RDW (%)	14.7 ± 2.7	14.6 ± 1.3	13.6 ± 1.6	13.2 ± 1.1
**Biochemistry**				
Ferritin (ng/mL)	35.9 ± 22.4	47.2 ± 37.9	88.1 ± 37.6	112.0 ± 71.3
Mild ID, ferritin < 30 ng/mL (%)	49.1	42.9	14.3	13.0
ID, ferritin < 15 ng/mL (%)	17.5	35.7	0.0	0.0
Iron (µg/dL)	100.2 ± 43.0	104.1 ± 45.2	110.9 ± 47.0	114.7 ± 41.0
Deficiency (%)	5.3	7.1	14.3	5.6

Parameters from students with low (<100) and high (≥100) recommended intake of iron (RI). Data are means ± standard deviation of indicated n participants. * *p* < 0.05, ** *p* < 0.01 and *** *p* < 0.001 significantly different from the corresponding “%RI < 100” group MCV, mean corpuscular volume; MCH, mean corpuscular hemoglobin; MCHC, mean corpuscular hemoglobin concentration; RDW, red cell distribution width; ID, iron deficiency. Iron deficiency, iron < 37 µg/dL (female) or 59 µg/dL (male).

**Table 7:** Blood parameters related to the recommended intake of iron and total intelligence quotient.

		**TIQ < 90**		**TIQ ≥ 90**	
		**<100% RI** **(n = 38)**	**≥100% RI** **(n = 7)**	**<100% RI** **(n = 19)**	**≥100% RI** **(n = 7)**
**FEMALE**	**HEMATOLOGY**				
	Erythrocyte (10^6^/μL)	4.7 ± 0.8	4.6 ± 0.3	4.7 ± 0.3	4.6 ± 0.2
	Hemoglobin (g/dL)	14.1 ± 2.4	13.3 ± 1.0	13.6 ± 0.9	13.8 ± 0.7
	Hematocrit (%)	41.8 ± 7.1	39.6 ± 2.7	40.6 ± 2.5	40.9 ± 2.3
	MCV (fL)	89.3 ± 14.7	86.8 ± 3.4	87.0 ± 6.4	89.7 ± 2.3
	MCH (pg)	30.1 ± 5.0	29.1 ± 1.4	29.3 ± 2.4	30.3 ± 0.9
	MCHC (%)	33.7 ± 5.4	33.5 ± 0.5	33.6 ± 0.5	33.7 ± 0.6
	RDW (%)	14.5 ± 2.5	15.3 ± 1.4	15.1 ± 3.0	13.9 ± 0.9 †
	**BIOCHEMISTRY**				
	Ferritin (ng/mL)	37.2 ± 21.8	26.7 ± 23.3	33.4 ± 24.0	67.6 ± 39.8 *,†
	Mild ID, ferritin < 30 ng/mL (%)	44.7	71.4	57.9	14.3
	ID, ferritin < 15 ng/mL (%)	15.8	57.1	21.1	14.3
	Iron (μg/dL)	102.1 ± 42.5	94.4 ± 56.3	96.3 ± 45.0	113.7 ± 32.0
	Iron deficiency (%)	5.3	14.3	5.3	0.0
		**TIQ < 90**		**TIQ ≥ 90**	
		**<100% RI** **(n = 2)**	**≥100% RI** **(n = 15)**	**<100% RI** **(n = 5)**	**≥100% RI** **(n = 39)**
**MALE**	**HEMATOLOGY**				
	Erythrocyte (10^6^/μL)	5.4 ± 0.2	5.3 ± 0.4	5.5 ± 0.3	5.4 ± 0.3
	Hemoglobin (g/dL)	15.9 ± 0.6	16.1 ± 0.9	16.7 ± 1.0	16.0 ± 0.9
	Hematocrit (%)	47.8 ± 1.2	47.6 ± 2.8	49.4 ± 3.6	47.5 ± 2.4
	MCV (fL)	88.9 ± 1.6	89.6 ± 2.6	89.4 ± 3.8	88.5 ± 3.2
	MCH (pg)	29.6 ± 0.3	30.3 ± 1.2	30.3 ± 1.3	29.8 ± 1.1
	MCHC (%)	33.3 ± 0.4	33.7 ± 0.8	33.9 ± 0.7	33.6 ± 0.7
	RDW (%)	13.5 ± 0.9	13.5 ± 1.0	13.7 ± 1.8	13.0 ± 1.1
	**BIOCHEMISTRY**				
	Ferritin (ng/mL)	125.3 ± 42.5	114.3 ± 70.4	73.2 ± 26.5	111.1 ± 72.6
	Mild ID, ferritin < 30 ng/mL (%)	0.0	13.3	20.0	12.8
	ID, ferritin < 15 ng/mL (%)	0.0	0.0	0.0	0.0
	Iron (μg/dL)	115.5 ± 71.4	122.6 ± 36.3	109.0 ± 45.0	111.7 ± 42.7
	Iron deficiency (%)	0.0	6.7	20.0	5.1

Data are means ± standard deviation of indicated n participants. * *p* < 0.05 significantly different from the corresponding “%RI < 100” group value. † *p* < 0.05 significantly different from the corresponding “<90” group. RI, recommended intake of iron; MCV, mean corpuscular volume; MCH, mean corpuscular hemoglobin; MCHC, mean corpuscular hemoglobin concentration; RDW, red cell distribution width; ID, iron deficiency. Iron deficiency, serum iron < 37 µg/dL (female) or 59 µg/dL (male).

**Table 8:** Multiple linear regression analysis of the association of TIQ with iron status related variables performed in female students.

GROUPS		β_0_	β_1_	β_2_	β_3_	β_4_
**FEMALE (overall)** R^2^ = 0.1119 n = 71		**Intercept**	**% RI**	**Hemoglobin (g/dL)**	**Iron (µg/dL)**	**Ferritin (ng/mL)**
	**Unstandardized estimate**	100.1	0.04412	−1.277	−0.02317	0.05324
	**95% CI**	67.94 to 132.2	0.001032 to 0.08721	−3.557 to 1.003	−0.07512 to 0.02877	−0.03237 to 0.1388
		**Intercept**	**%RI (z−score)**	**Hemoglobin (z−score)**	**Iron (z−score)**	**Ferritin (z−score)**
	**Standardized estimate**	85.77	1.926	−1.096	−0.9678	1.385
	**95% CI**	83.96 to 87.59	0.04504 to 3.806	−3.054 to 0.8611	−3.138 to 1.202	−0.8421 to 3.612
	***p*** **−value**	<0.0001	0.0449	0.2675	0.3764	0.2187
**TIQ < 90** R^2^ = 0.04305 n = 45		**Intercept**	**% RI**	**Hemoglobin (g/dL)**	**Iron (µg/dL)**	**Ferritin (ng/mL)**
	**Unstandardized estimate**	77.47	−0.006827	0.4591	−0.008289	−0.04401
	**95% CI**	47.26 to 107.7	−0.06260 to 0.04894	−1.628 to 2.546	−0.05005 to 0.03347	−0.1321 to 0.04406
		**Intercept**	**% RI (z−score)**	**Hemoglobin (z−score)**	**Iron (z−score)**	**Ferritin (z−score)**
	**Standardized estimate**	80.75	−0.298	0.3941	−0.3462	−1.145
	**95% CI**	79.12 to 82.38	−2.732 to 2.136	−1.397 to 2.186	−2.091 to 1.398	−3.436 to 1.146
	***p*** **−value**	<0.0001	0.8059	0.659	0.6904	0.3186
**TIQ ≥ 90** R^2^ = 0.32 n = 26		**Intercept**	**% RI**	**Hemoglobin (g/dL)**	**Iron (µg/dL)**	**Ferritin (ng/mL)**
	**Unstandardized estimate**	100	0.03271	−0.5735	−0.02359	0.0274
	**95% CI**	75.18 to 124.9	0.007172 to 0.05824	−2.403 to 1.256	−0.07187 to 0.02469	−0.03611 to 0.09092
		**Intercept**	**% RI (z−score)**	**Hemoglobin (z−score)**	**Iron (z−score)**	**Ferritin (z−score)**
	**Standardized estimate**	93.53	1.428	−0.4924	−0.9852	0.713
	**95% CI**	91.98 to 95.07	0.3130 to 2.542	−2.063 to 1.078	−3.002 to 1.032	−0.9395 to 2.365
	***p*** **−value**	<0.0001	0.0145	0.5215	0.3212	0.3798
**%RI < 100** R^2^ = 0.112 n = 57		**Intercept**	**% RI**	**Hemoglobin (g/dL)**	**Iron (µg/dL)**	**Ferritin (ng/mL)**
	**Unstandardized estimate**	111.6	0.1008	−2.221	−0.01925	−0.005461
	**95% CI**	80.21 to 143.0	−0.03154 to 0.2332	−4.561 to 0.1195	−0.07041 to 0.03190	−0.1049 to 0.09395
		**Intercept**	**% RI (z−score)**	**Hemoglobin (z−score)**	**Iron (z−score)**	**Ferritin (z−score)**
	**Standardized estimate**	87.18	4.401	−1.907	−0.8043	−0.1421
	**95% CI**	84.26 to 90.10	−1.377 to 10.18	−3.916 to 0.1026	−2.941 to 1.333	−2.728 to 2.444
	***p*** **−value**	<0.0001	0.1324	0.0624	0.4535	0.9126
**%RI ≥ 100** R^2^ = 0.6387 n = 14		**Intercept**	**% RI**	**Hemoglobin (g/dL)**	**Iron (µg/dL)**	**Ferritin (ng/mL)**
	**Unstandardized estimate**	31.55	0.1423	2.129	−0.03486	0.1948
	**95% CI**	−70.11 to 133.2	0.04026 to 0.2443	−5.227 to 9.485	−0.2035 to 0.1338	0.001661 to 0.3880
		**Intercept**	**% RI (z−score)**	**Hemoglobin (z−score)**	**Iron (z−score)**	**Ferritin (z−score)**
	**Standardized estimate**	76.98	6.21	1.827	−1.456	5.069
	**95% CI**	68.55 to 85.41	1.757 to 10.66	−4.488 to 8.142	−8.501 to 5.589	0.04321 to 10.10
	***p*** **−value**	0.5005	0.0116	0.5291	0.6513	0.0484
**TIQ < 90** **%RI < 100** R^2^ = 0.05356 n = 38		**Intercept**	**% RI**	**Hemoglobin (g/dL)**	**Iron (µg/dL)**	**Ferritin (ng/mL)**
	**Unstandardized estimate**	78.8	0.03312	0.1907	−0.009043	−0.03565
	**95% CI**	47.06 to 110.5	−0.08951 to 0.1558	−2.081 to 2.462	−0.05323 to 0.03514	−0.1270 to 0.05565
		**Intercept**	**% RI (z−score)**	**Hemoglobin (z−score)**	**Iron (z−score)**	**Ferritin (z−score)**
	**Standardized estimate**	81.97	1.446	0.1637	−0.3777	−0.9276
	**95% CI**	79.20 to 84.73	−3.907 to 6.799	−1.786 to 2.114	−2.223 to 1.468	−3.303 to 1.448
	***p*** **−value**	<0.0001	0.5863	0.8654	0.6798	0.4326
**TIQ < 90** **%RI ≥ 100** R2 = 0.9707 n = 7		**Intercept**	**% RI**	**Hemoglobin (g/dL)**	**Iron (µg/dL)**	**Ferritin (ng/mL)**
	**Unstandardized estimate**	40.78	0.1883	0.5949	0.09269	−0.1822
	**95% CI**	−14.88 to 96.44	0.07538 to 0.3013	−3.078 to 4.268	−0.005881 to 0.1913	−0.4032 to 0.03889
		**Intercept**	**% RI (z−score)**	**Hemoglobin (z−score)**	**Iron (z−score)**	**Ferritin (z−score)**
	**Standardized estimate**	67.35	8.221	0.5107	3.872	−4.74
	**95% CI**	60.91 to 73.79	3.290 to 13.15	−2.642 to 3.664	−0.2457 to 7.989	−10.49 to 1.012
	***p*** **−value**	0.0876	0.0189	0.558	0.056	0.0712
**TIQ ≥ 90** **%RI < 100** R^2^ = 0.2247 n = 19		**Intercept**	**% RI**	**Hemoglobin (g/dL)**	**Iron (µg/dL)**	**Ferritin (ng/mL)**
	**Unstandardized estimate**	107.5	0.1166	−1.505	−0.02436	0.01431
	**95% CI**	78.71 to 136.2	−0.01943 to 0.2526	−3.905 to 0.8948	−0.07744 to 0.02872	−0.08320 to 0.1118
		**Intercept**	**% RI (z−score)**	**Hemoglobin (z−score)**	**Iron (z−score)**	**Ferritin (z−score)**
	**Standardized estimate**	94.55	5.088	−1.292	−1.018	0.3724
	**95% CI**	92.05 to 97.04	−0.8479 to 11.02	−3.353 to 0.7682	−3.235 to 1.200	−2.165 to 2.909
	***p*** **−value**	<0.0001	0.0873	0.2	0.3417	0.7576
**TIQ ≥ 90** **%RI ≥ 100** R^2^ = 0.4673 n = 7		**Intercept**	**% RI**	**Hemoglobin (g/dL)**	**Iron (µg/dL)**	**Ferritin (ng/mL)**
	**Unstandardized estimate**	128.7	−0.01839	−0.8551	−0.211	0.1014
	**95% CI**	−263.6 to 521.0	−0.3953 to 0.3586	−24.84 to 23.13	−1.310 to 0.8877	−0.4680 to 0.6707
		**Intercept**	**% RI (z−score)**	**Hemoglobin (z−score)**	**Iron (z−score)**	**Ferritin (z−score)**
	**Standardized estimate**	97.86	−0.8026	−0.7341	−8.812	2.637
	**95% CI**	60.17 to 135.5	−17.26 to 15.65	−21.32 to 19.86	−54.70 to 37.08	−12.18 to 17.45
	***p*** **−value**	0.2936	0.8532	0.8922	0.4956	0.5238

Unstandardized (in the corresponding independent variable units) and standardized (in standard deviation units, z-score) ß-coefficients and 95% CI obtained in the multiple linear regression analysis performed in female students (grouped as indicated, according to %RI and TIQ value range). CI: confidence intervals.

**Table 9:** Multiple linear regression analysis of the association of TIQ with iron status related variables performed in male students.

GROUPS		β_0_	β_1_	β_2_	β_3_	β_4_
**MALE (overall)** R^2^ = 0.02492 n = 58		**Intercept**	**% RI**	**Hemoglobin (g/dL)**	**Iron (µg/dL)**	**Ferritin (ng/mL)**
	**Unstandardized estimate**	98.73	−0.018	0.2229	−0.008961	−0.02229
	**95% CI**	38.25 to 159.2	−0.08071 to 0.04470	−3.499 to 3.945	−0.09718 to 0.07926	−0.06842 to 0.02384
		**Intercept**	**% RI (z−score)**	**Hemoglobin (z−score)**	**Iron (z−score)**	**Ferritin (z−score)**
	**Standardized estimate**	96.19	−0.9055	0.192	−0.3298	−1.547
	**95% CI**	93.13 to 99.25	−4.059 to 2.248	−3.014 to 3.398	−3.577 to 2.917	−4.748 to 1.654
	***p*** **−value**	0.0019	0.5671	0.9049	0.8393	0.3368
**TIQ < 90** R^2^ = 0.2877 n = 17		**Intercept**	**% RI**	**Hemoglobin (g/dL)**	**Iron (µg/dL)**	**Ferritin (ng/mL)**
	**Unstandardized estimate**	83.12	−0.02122	0.5637	−0.04979	0.003212
	**95% CI**	48.03 to 118.2	−0.06015 to 0.01771	−1.666 to 2.794	−0.1066 to 0.007063	−0.02860 to 0.03502
		**Intercept**	**% RI (z−score)**	**Hemoglobin (z−score)**	**Iron (z−score)**	**Ferritin (z−score)**
	**Standardized estimate**	83.79	−1.067	0.4856	−1.833	0.2229
	**95% CI**	81.82 to 85.75	−3.025 to 0.8905	−1.435 to 2.407	−3.925 to 0.2599	−1.984 to 2.430
	***p*** **−value**	0.0002	0.2579	0.5919	0.0806	0.8295
**TIQ ≥ 90** R^2^ = 0.08355 n = 41		**Intercept**	**% RI**	**Hemoglobin (g/dL)**	**Iron (µg/dL)**	**Ferritin (ng/mL)**
	**Unstandardized estimate**	107.6	−0.001585	−0.706	0.06816	−0.01536
	**95% CI**	50.93 to 164.2	−0.06043 to 0.05726	−4.155 to 2.743	−0.01602 to 0.1523	−0.05664 to 0.02592
		**Intercept**	**% RI (z−score)**	**Hemoglobin (z−score)**	**Iron (z−score)**	**Ferritin (z−score)**
	**Standardized estimate**	101.9	−0.07972	−0.6082	2.509	−1.066
	**95% CI**	99.09 to 104.7	−3.039 to 2.880	−3.579 to 2.363	−0.5895 to 5.607	−3.930 to 1.799
	***p*** **−value**	0.0005	0.9567	0.6805	0.1093	0.4554
**%RI < 100** R^2^ = 0.2705 n = 7		**Intercept**	**% RI**	**Hemoglobin (g/dL)**	**Iron (µg/dL)**	**Ferritin (ng/mL)**
	**Unstandardized estimate**	88.18	−0.2856	2.96	0.03285	−0.1766
	**95% CI**	−669.8 to 846.1	−3.262 to 2.691	−38.39 to 44.31	−0.8644 to 0.9301	−1.228 to 0.8749
		**Intercept**	**% RI (z−score)**	**Hemoglobin (z−score)**	**Iron (z−score)**	**Ferritin (z−score)**
	**Standardized estimate**	77	−14.36	2.55	1.209	−12.26
	**95% CI**	−121.3 to 275.3	−164.1 to 135.3	−33.07 to 38.17	−31.82 to 34.23	−85.22 to 60.71
	***p*** **−value**	0.6663	0.7198	0.7872	0.8893	0.545
**%RI ≥ 100** R^2^ = 0.01318 n = 51		**Intercept**	**% RI**	**Hemoglobin (g/dL)**	**Iron (µg/dL)**	**Ferritin (ng/mL)**
	**Unstandardized estimate**	102.3	−0.001139	−0.2044	−0.01506	−0.01424
	**95% CI**	38.25 to 166.4	−0.07247 to 0.07019	−4.253 to 3.844	−0.1124 to 0.08225	−0.06094 to 0.03246
		**Intercept**	**% RI (z−score)**	**Hemoglobin (z−score)**	**Iron (z−score)**	**Ferritin (z−score)**
	**Standardized estimate**	95.68	−0.05727	−0.1761	−0.5544	−0.9881
	**95% CI**	92.42 to 98.94	−3.645 to 3.530	−3.663 to 3.311	−4.136 to 3.027	−4.229 to 2.252
	***p*** **−value**	0.0024	0.9745	0.9195	0.7568	0.5424
**TIQ < 90** **%RI < 100** R^2^ = 0 n = 2		**Intercept**	**% RI**	**Hemoglobin (g/dL)**	**Iron (µg/dL)**	**Ferritin (ng/mL)**
	**Unstandardized estimate**	0	0	0	0	0
	**95% CI**	0	0	0	0	0
		**Intercept**	**% RI (z−score)**	**Hemoglobin (z−score)**	**Iron (z−score)**	**Ferritin (z−score)**
	**Standardized estimate**	0	0	0	0	0
	**95% CI**	0	0	0	0	0
	***p*** **−value**	0	0	0	0	0
**TIQ < 90** **%RI ≥ 100** R^2^ = 0.1921 n = 15		**Intercept**	**% RI**	**Hemoglobin (g/dL)**	**Iron (µg/dL)**	**Ferritin (ng/mL)**
	**Unstandardized estimate**	81.88	−0.01621	0.5568	−0.04543	0.002051
	**95% CI**	41.49 to 122.3	−0.06965 to 0.03723	−1.938 to 3.052	−0.1194 to 0.02856	−0.03412 to 0.03822
		**Intercept**	**% RI (z−score)**	**Hemoglobin (z−score)**	**Iron (z−score)**	**Ferritin (z−score)**
	**Standardized estimate**	83.56	−0.8151	0.4797	−1.672	0.1423
	**95% CI**	80.95 to 86.17	−3.503 to 1.872	−1.669 to 2.629	−4.395 to 1.051	−2.367 to 2.652
	***p*** **−value**	0.0011	0.5145	0.6297	0.2012	0.902
**TIQ ≥ 90** **%RI < 100** R^2^ = 1 n = 5		**Intercept**	**% RI**	**Hemoglobin (g/dL)**	**Iron (µg/dL)**	**Ferritin (ng/mL)**
	**Unstandardized estimate**	−391.8	3.285	−0.07885	0.9341	1.571
	**95% CI**	0	0	0	0	0
		**Intercept**	**% RI (z−score)**	**Hemoglobin (z−score)**	**Iron (z−score)**	**Ferritin (z−score)**
	**Standardized estimate**	378.1	165.2	−0.06792	34.38	109
	**95% CI**	0	0	0	0	0
	***p*** **−value**	0	0	0	0	0
**TIQ ≥ 90** **%RI ≥ 100** R^2^ = 0.1151 n = 36		**Intercept**	**% RI**	**Hemoglobin (g/dL)**	**Iron (µg/dL)**	**Ferritin (ng/mL)**
	**Unstandardized estimate**	113.1	0.01916	−1.319	0.06772	−0.01067
	**95% CI**	53.65 to 172.6	−0.04525 to 0.08358	−5.067 to 2.429	−0.02419 to 0.1596	−0.05083 to 0.02950
		**Intercept**	**% RI (z−score)**	**Hemoglobin (z−score)**	**Iron (z−score)**	**Ferritin (z−score)**
	**Standardized estimate**	101.2	0.9638	−1.136	2.492	−0.7402
	**95% CI**	98.40 to 103.9	−2.276 to 4.203	−4.365 to 2.092	−0.8903 to 5.875	−3.527 to 2.047
	***p*** **−value**	0.0005	0.5484	0.4783	0.143	0.5919

Unstandardized (in the corresponding independent variable units) and standardized (in standard deviation units, z-score) ß-coefficients and 95% CI obtained in the multiple linear regression analysis performed in female students (grouped as indicated, according to %RI and TIQ value range). CI: confidence intervals.

## Data Availability

Data is contained within the article.
